# Evidence against a Beneficial Effect of Irisin in Humans

**DOI:** 10.1371/journal.pone.0073680

**Published:** 2013-09-11

**Authors:** Silja Raschke, Manuela Elsen, Hans Gassenhuber, Mark Sommerfeld, Uwe Schwahn, Barbara Brockmann, Raphael Jung, Ulrik Wisløff, Arnt E. Tjønna, Truls Raastad, Jostein Hallén, Frode Norheim, Christian A. Drevon, Tania Romacho, Kristin Eckardt, Juergen Eckel

**Affiliations:** 1 Paul-Langerhans-Group, Integrative Physiology, German Diabetes Center, Düsseldorf, Germany; 2 R&D Diabetes Division, Sanofi-Aventis Deutschland GmbH, Frankfurt, Germany; 3 K.G. Jebsen Center of Exercise in Medicine at Department of Circulation and Medical Imaging, Norwegian University of Science and Technology, Trondheim, Norway; 4 Norwegian School of Sport Sciences, Oslo, Norway; 5 Department of Nutrition, Institute of Basic Medical Sciences, Faculty of Medicine, University of Oslo, Oslo, Norway; Universidad Pablo de Olavide, Centro Andaluz de Biología del Desarrollo-CSIC, Spain

## Abstract

Brown adipose tissue has gained interest as a potential target to treat obesity and metabolic diseases. Irisin is a newly identified hormone secreted from skeletal muscle enhancing browning of white fat cells, which improves systemic metabolism by increasing energy expenditure in mice. The discovery of irisin raised expectations of its therapeutic potential to treat metabolic diseases. However, the effect of irisin in humans is unclear. Analyses of genomic DNA, mRNA and expressed sequence tags revealed that *FNDC5*, the gene encoding the precursor of irisin, is present in rodents and most primates, but shows in humans a mutation in the conserved start codon ATG to ATA. HEK293 cells transfected with a human FNDC5 construct with ATA as start codon resulted in only 1% full-length protein compared to human *FNDC5* with ATG. Additionally, *in vitro* contraction of primary human myotubes by electrical pulse stimulation induced a significant increase in PGC1α mRNA expression. However, *FNDC5* mRNA level was not altered. *FNDC5* mRNA expression in muscle biopsies from two different human exercise studies was not changed by endurance or strength training. Preadipocytes isolated from human subcutaneous adipose tissue exhibited differentiation to brite human adipocytes when incubated with bone morphogenetic protein (BMP) 7, but neither recombinant FNDC5 nor irisin were effective. In conclusion, our findings suggest that it is rather unlikely that the beneficial effect of irisin observed in mice can be translated to humans.

## Introduction

Obesity and the involved risk of developing metabolic diseases represent a major global public health challenge. In obese patients glucose homeostasis is disturbed due to an imbalance between energy intake and energy expenditure. Although the understanding of the role of genetics in obesity and type 2 diabetes is increasing [1]–[3], roughly 60% of all cases of diabetes can be directly attributed to weight gain [4].

Brown adipose tissue (AT) has drawn attention as a novel preventive and therapeutic target to treat obesity and metabolic diseases like type 2 diabetes. Whereas white AT is the primary site of triglyceride storage, brown AT is specialized in energy expenditure. In order to maintain body temperature in a cold environment, brown AT oxidizes fatty acids and generates heat [5] by the mitochondrial uncoupling protein 1 (UCP1). Thus, UCP1 knock-out mice are cold sensitive and tend to develop obesity, even when fed a control diet [6], whereas experimental approaches aiming to increase the amount and activity of brown AT reduce the development of obesity [7]. Brown AT has also been detected in humans and is found in anatomically discrete depots, with the most common location in adults in the cervical-supraclavicular depot [8]–[12].

Brown adipocytes and skeletal muscle cells arise from progenitors expressing myf5 [13] and their differentiation is specifically controlled by transcriptional regulators like *PRDM16* [14], *PGC1α* [15], and others [16]–[19]. Chronical stimulation of mouse preadipocytes derived from epididymal white AT with rosiglitazone, a *PPARγ* agonist, reveals a thermogenic competent population of UCP1-expressing adipocytes [20]. These cells do not represent classical brown adipocytes, because they do not express typical brown AT transcription factors such as *ZIC1* and *PRDM16.* Instead, these cells appear to be a particular type of adipocytes termed as ‘brite’ (brown-in-white) adipocytes. Thus, the possibility to switch from white AT to brite AT and to identify mechanisms that can activate white to brown trans-differentiation in response to pharmacological compounds is highly attractive in the context of obesity treatment.

Boström *et al.* published a promising mechanism for the induction of brite adipocytes in white AT depots after exercise in mice. Overexpression of *PGC1α* in mice skeletal muscle as well as exercise induced expression of the *FNDC5* gene [21], a gene which has scarcely been studied before. In 2002 two different groups first described the mouse sequence of *FNDC5* [22], [23]. In adult murine tissues, *FNDC5* is highly expressed in heart and brain and less in skeletal muscle [22], [23]. FNDC5 is described as a protein containing a signal peptide, fibronectin type III repeats, and hydropathy analysis revealed a hydrophobic region, which is likely to encode a transmembrane domain. Previous studies linked the gene to differentiation of myoblasts and neurones [23], [24], and it has been suggested that FNDC5 is located in the matrix of peroxisomes [23]. However, Boström *et al.* showed that the transmembrane protein is cleaved by transfected HEK293 cells and the extracellular part of the protein is released, which acts as novel molecule called irisin [21]. Viral delivery of *FNDC5* in mice caused browning of subcutaneous fat, stimulated oxygen consumption, and diminished diet-induced weight gain and metabolic dysfunction [21]. Thus, irisin induced a thermogenic mechanism in white AT, which improved whole body energy balance in mice. This initial report of irisin linked the *FNDC5* gene to browning in mice.

Furthermore, Boström *et al*. were the first to describe this gene in humans [21]. The bioinformatics analysis of the *FNDC5* gene performed by us revealed that divergent sequences have been published. Until the protein sequence was modified September 5, 2012 the UniProt database entry FNDC5/Q8NAU1 represented the full-length protein as described by Boström *et al.* [21]. The UniProt entry was modified, since the underlying transcript sequence was classified as artefact. Now, two potential protein sequences are available at the UniProt database. Ivanov *et al*. described human FNDC5 as a gene with a mutation in the start codon to ATA [25], encoding isoleucin, instead of ATG, encoding methionine. Using this non-canonical start site would generate the full-length protein. The translation of the second protein sequence starts at the first in-frame ATG start codon of the FNDC5 ORF, but as this ATG is located 76 codons downstream the resulting protein would be a truncated irisin protein.

Although, the initial description of irisin was focused on mice, Boström *et al*. raised the hope that exogenously administered irisin might have a therapeutic potential in the treatment of obesity and diabetes in humans. Thus, the aim of our present study was to analyze the human *FNDC5* gene and to explore its function in the human system. Our findings indicate that great caution is needed when extrapolating data regarding FNDC5/irisin from rodent to the human situation.

## Results

### Start Codon of FNDC5 Gene is Mutated in Humans

**Figure 1:**
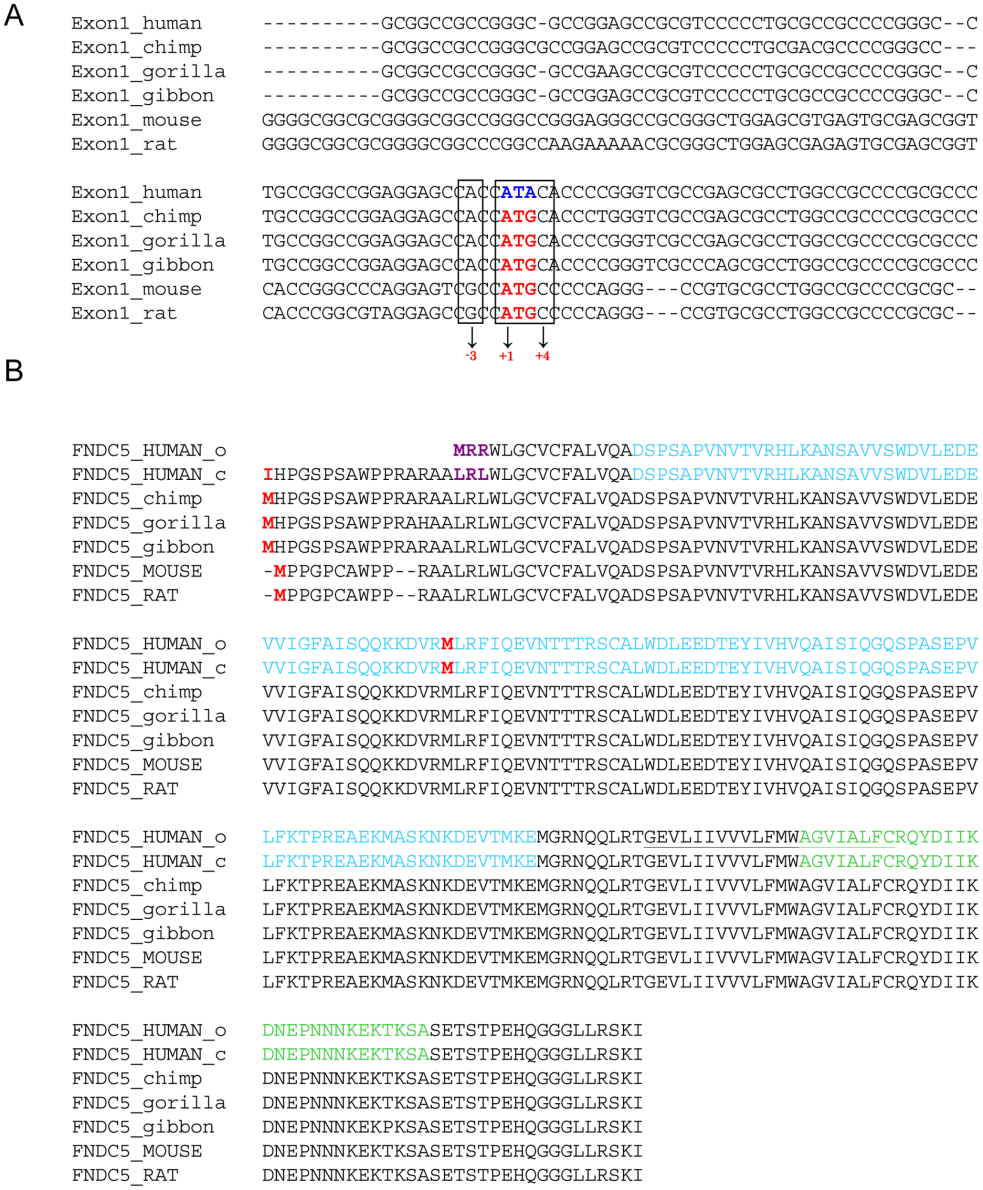
The human FNDC5 gene differs from other species by a mutation in the start codon. (A) Multiple alignment of the exon 1 sequences: the conserved partial Kozak ATG start sequence of FNDC5 is bold and red. The mutated ATG to ATA in human is bold and blue. There is no other ATG present in exon 1. (B) Multiple sequence alignment of FNDC5 proteins of different species including two human versions. FNDC5_human_o: sequence published by Boström et al.; FNDC5_human_c: current version in Uniprot; red M = start methionines including the potential downstream human start site; light blue = irisin sequence; blue I = mutated start site claimed to be a non canonical start site; purple LRL = sequence shown in UniProt (Q8NAU1_old) as MRR. The underlined sequence indicates the transmembrane part of the protein. Green sequence = peptide used for the generation of the Abcam FNDC5 antibody.

A multi-species sequence alignment of the *FNDC5* exon 1 demonstrated that *FNDC5* genes from different species like rat, mouse, gibbon, gorilla and chimp display a conserved ATG translation start site, except for the human sequence (Figure 1A). In contrast, at the position of the start codon the human sequence shows an ATA, encoding isoleucin (I), instead of the conserved ATG, encoding methionine (M). Using 5′-RACE-PCR, we could confirm experimentally the mutation within the start codon as ATA in human brain and skeletal muscle samples (Figure S1). According to the current UniProt entry Q8NAU1 (Figure 1B, FNDC5_human_c) this is potentially a non-canonical start site and could still produce to full-length FNDC5, which might be later proteolytically cleaved to release irisin.

Before translation start, the ribosome recognizes a conserved mRNA sequence as the start site for protein translation, called Kozak consensus sequence (GCCXCCATGG, X = A or G). However, Kozak *et al.* showed that a mutated start codon to ATA, even in a perfect context (GCCXCCATAG, X = A or G, Figure 1A), was highly unlikely to serve as a translation site and resulted in low translation efficiency [26]. The next in-frame downstream ATG (M, marked in red in Figure 1B) is a non-Kozak ATG and located within the sequence that was annotated as irisin (irisin sequence marked in blue, Figure 1B). Thus, an N-terminal truncated *FNDC5* (represented by cDNA sequences NP_ 715637/NM_153756) and truncated irisin would be generated. In addition there are three upstream partial Kozak ATGs in this mRNA that are not in frame with the *FNDC5* open reading frame (ORF) and would therefore strongly reduce translation from this new start site (Figure S3). It has been experimentally shown that the translation efficiency of non-canonical sites can be increased, if a hairpin slows down the scanning ribosome [27], as described for *FGF2* (Figure S4A). Based on this observation, an ATG hairpin program predicts if there are stem-loop structures in an appropriate distance to the ATA [28], which would increase the translation efficiency. However, for human *FNDC5* no eligible hairpin structures were found (Figure S4B).

A comparison of the full-length human FNDC5 protein sequence published by Boström *et al.* (Figure 1B, FNDC5_human_o, [21]) with mRNA, expressed sequence tags, genomic DNA and single-nucleotide polymorphism data revealed that the first three amino acids (MRR, Figure 1B) do not match human genomic DNA. We excluded that this is due to differential splicing, because 20 expressed sequence tags and two Ref_Seq cDNAs that cover this region, perfectly matched the annotated exon 1 region (Figure S2). We analyzed public single-nucleotide polymorphism data, but could not find a reference that this codon might be altered.

### Full-length Human FNDC5 with ATA as Start Codon is not Translated into Protein

Nevertheless, *FNDC5* mRNA is expressed in human tissues, predominantly in the heart, less in muscle and brain (Figure S5). In certain cases, translation initiation can occur at codons differing from ATG by a single nucleotide, like ATA [25]. To verify whether human FNDC5 was translated into full-length protein with a non-ATG initiation, the human gene was cloned into the expression vector pcDNA5-FRT TO-cEGFP. In addition to this vector with ATA as start codon (ATA-hFNDC5-GFP), a second vector with ATG as classical start codon (ATG-hFNDC5-GFP) (Figure S6) and as a control murine FNDC5 (mFNDC5-GFP) was cloned. All vectors were transfected into HEK293 cells.

Although transient transfection of HEK293 cells with the expression vectors of mFNDC5-GFP and ATG-hFNDC5-GFP resulted in a clearly detectable fluorescence signal due to the expression of GFP-FNDC5 fusion protein, most importantly, for ATA-hFNDC5-GFP transfected cells the signal was hardly detectable (Figure S7A, B).

**Figure 2:**
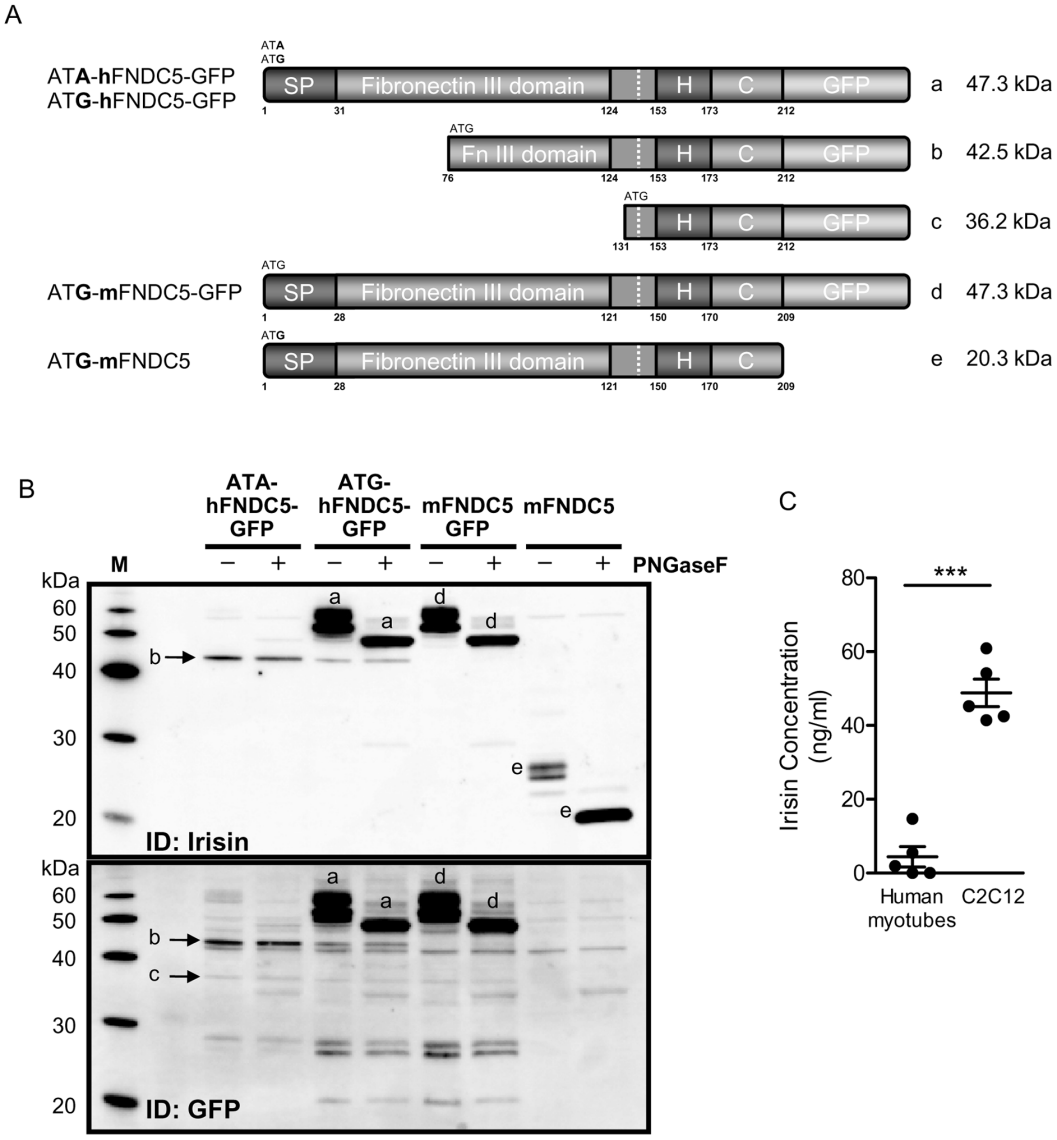
Human FNDC5 with an ATA start codon is translated into full-length protein only at very low abundance. (A) Schematic representation of the predicted FNDC5 protein structures. Using the first ATG/ATA as start codon of human FNDC5 tagged with GFP would result in full-length FNDC5 protein (a). The use of downstream ATG as start codon would result in truncated FNDC5 protein isoforms (b and c). Murine FNDC5 with ATG as start codon tagged with GFP (d) or without GFP (e). (B) Expression of FNDC5 in HEK293 cells. Cells transfected with constructs containing human FNDC5-GFP gene with ATA and ATG as start codon as well as mouse FNDC5-GFP gene were analyzed 24 h after transfection. Cell lysates were analyzed by immunodetection using antibodies against irisin/FNDC5 and GFP. Cell lysates were treated with PNGaseF to deglycosylate proteins. (C) Supernatants of primary human and C2C12 myotubes were collected for 24 h in serum-free medium and concentrated 60fold using centrifugal filter devices. Irisin protein levels in concentrated supernatants were measured using EIA kit. Medium alone showed no cross-reactivity with the kit; n = 5, *** p<0.001.

Analysing the protein level of transfected HEK293 cells revealed that the human construct with ATG as start codon produced similar amounts of full-length protein compared to murine FNDC5. The protein can be detected in two distinct bands of 52 and 56 kDa as shown by western blot analysis (Figure 2B). Full-length FNDC5 protein seems to be glycosylated, because incubation of cell lysates with N-glycosidase F (PNGase F) resulted in merging of the two bands into one signal with a significantly decreased size of 48 kDa. In contrast, the human transcript with ATA as start codon resulted only in 1% full-length protein as compared to ATG-hFNDC5-GFP. Instead the downstream in-frame ATG (represented by NP_715637, starting with MLRFIQEVN, Figure 2A (b)) was translated into a protein missing the first 76 amino acids. However, this ATG was used with strongly reduced efficiency and was apparently not glycosylated.

Additionally, at a molecular size of 36 kDa a very weak band was detected possibly representing the second downstream ATG of human FNDC5 (aa131) or murine FNDC5 (aa128), (Figure 2A (c), starting with MASKNKDE, Figure 1B).

A comparison of the irisin protein levels released by differentiated primary human skeletal muscle cells compared to murine C2C12 cells showed substantially lower irisin protein levels in the supernatant of human cells compared to murine cells (Figure 2C).

### FNDC5 Gene is not Activated by Contraction in Humans

**Figure 3:**
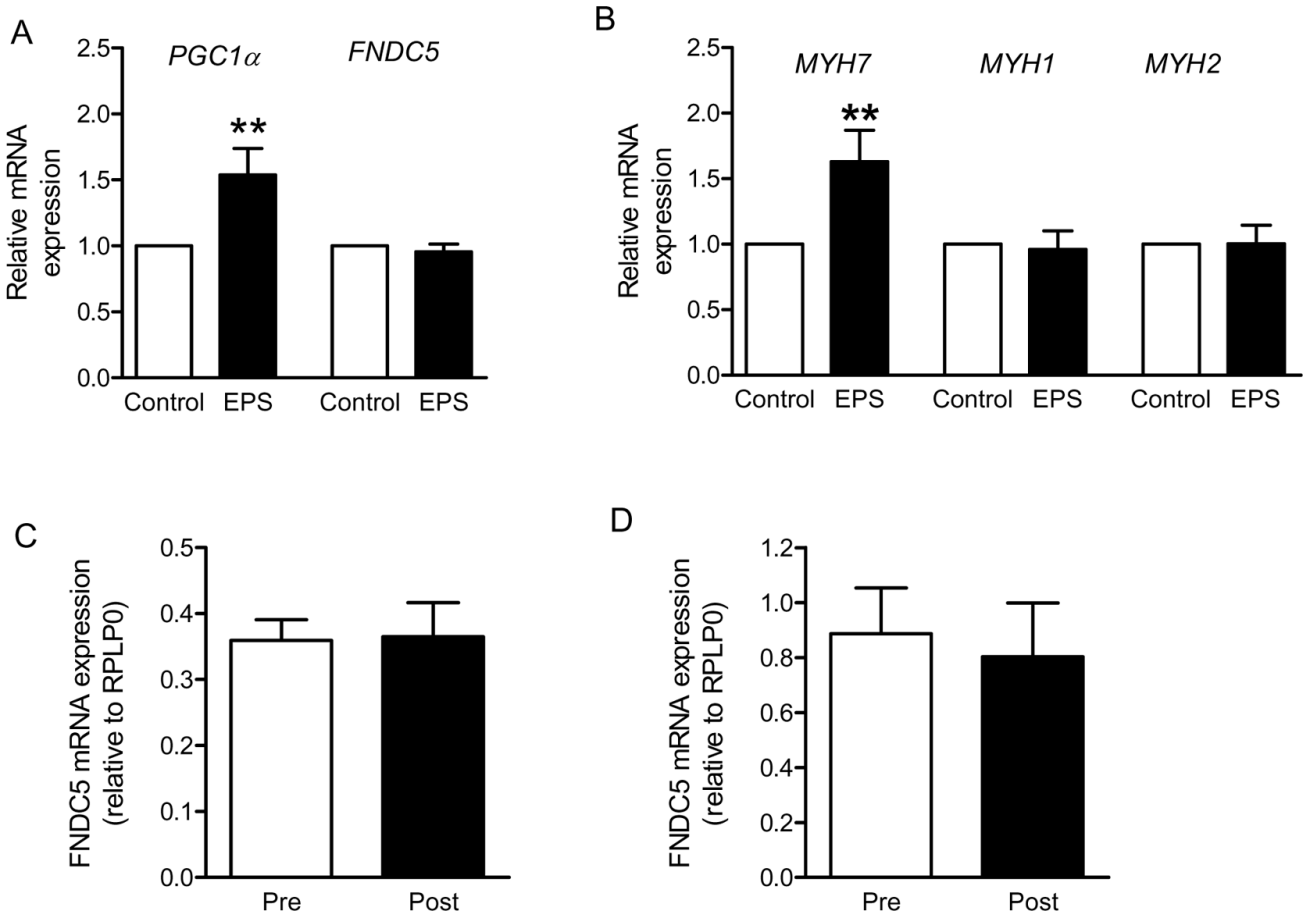
FNDC5 mRNA level is not contraction-regulated in skeletal muscle cells and is not increased by endurance or strength training in humans. (A) and (B) Primary human skeletal muscle cells were differentiated in αMEM containing 2% (vol./vol.) horse serum, followed by overnight starvation, and subjected to EPS for 24 h in serum-free medium (1 Hz, 2 ms, 11.5 V). Relative gene expression of *PGC1α*, *FNDC5* (A), *MYH1*, *2*, and *7* (B) was measured by quantitative real-time PCR (qRT-PCR). All expression data were normalized to actin; n = 5 (A), n = 10 (B); **p<0.01. White bars, control (non-EPS); black bars, EPS. (C) qRT-PCR analysis of *FNDC5* expression in *m. vastus lateralis* from young sedentary males before (Pre) and after 10 weeks (Post) of aerobic interval training (n = 6). (D) qRT-PCR analysis of *FNDC5* expression in *m. trapezius* from sedentary males before (Pre) and after 11 weeks (Post) of strength training (n = 7). All expression data were normalized to RPLP0. Data are presented as mean values ± SEM.

*PGC1α* gene expression is induced in muscle by exercise [29] and *FNDC5* gene expression was reported to be *PGC1α*-dependent in mice [21]. To study contraction-regulated gene expression, we previously developed an *in vitro* contraction model using electrical pulse stimulation (EPS) of primary human skeletal muscle cells [30]. By using this EPS model, *PGC1α* mRNA expression was significantly enhanced after 24 h of EPS in primary human skeletal muscle cells (1.5fold, Figure 3A). However, *FNDC5* mRNA expression was not altered (Figure 3A). This EPS-protocol, induced a significant upregulation of *MYH7* mRNA level (encoding myosin heavy chain (MHC) isoform 1 protein, 1.6fold), while *MYH2* (encoding MHC2a) and *MYH1* (encoding MHC2×) were unaltered (Figure 3B).

The gene expression of FNDC5 in human muscle biopsies was examined before and after extensive and documented training in two different cohorts. We found no FNDC5 gene activation by neither 10 weeks of interval endurance training among 41±2 years old males (Figure 3C) nor 11 weeks of strength training in 28±4 years old males with normal body weight (Figure 3D).

### Recombinant FNDC5 and Irisin have No Effect on the Brite Differentiation of Human Preadipocytes

We isolated preadipocytes from primary human subcutaneous AT and differentiated these cells to mature adipocytes in the presence of recombinant FNDC5 (200 ng/ml), irisin (60 ng/ml) or BMP7 (50 ng/ml) as a positive control, respectively. FNDC5 was obtained from Abnova, which also was used by Boström *et al.* [21] and Wu *et al.* [31]. In addition, we used recombinant FNDC5 protein obtained from Phoenix.

**Figure 4:**
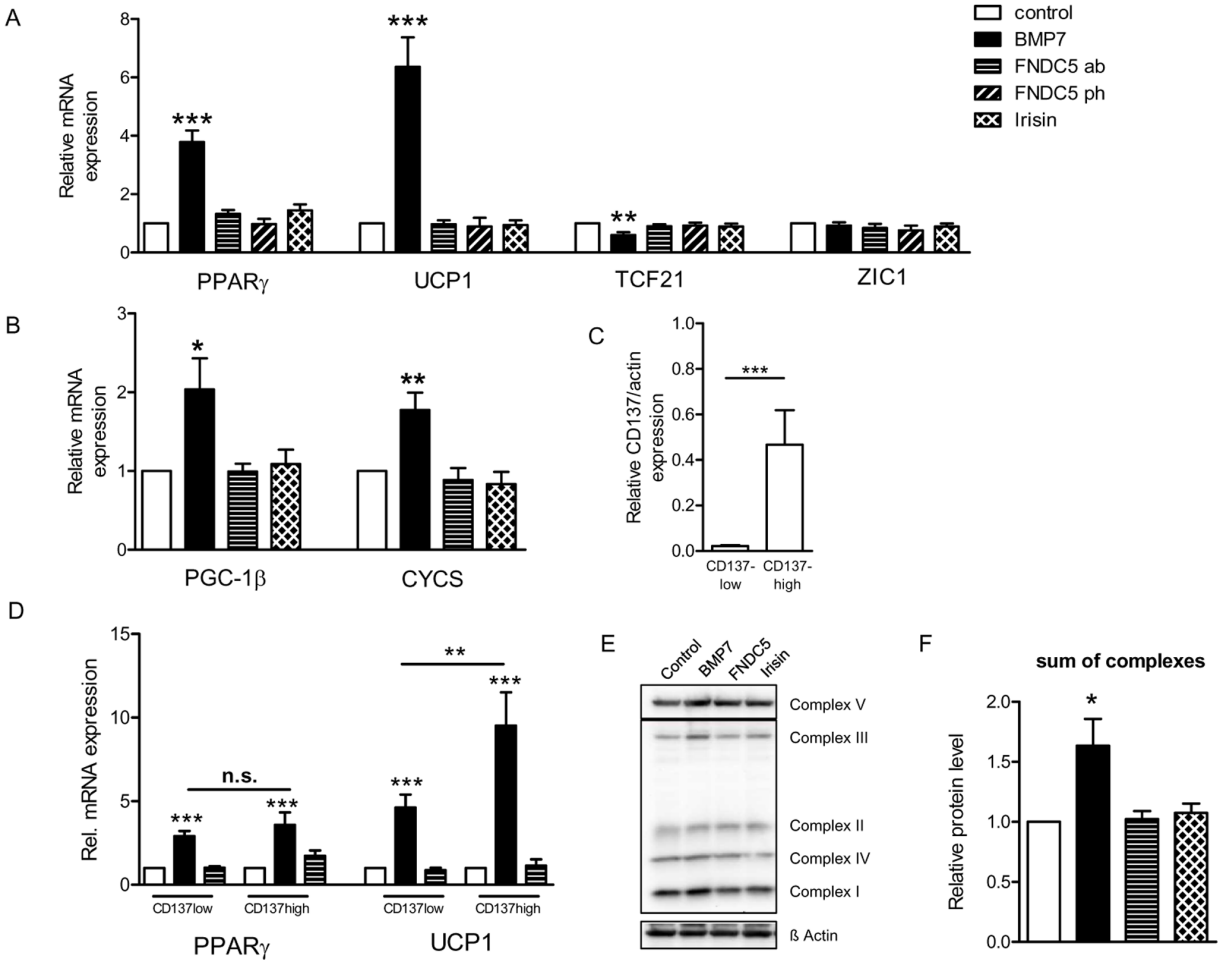
BMP7 activates the brite fat gene program in human adipocytes, but not FNDC5 and irisin. Isolated preadipocytes from human subcutaneous preadipocytes of different donors were differentiated in the presence of 50/ml BMP7, 200 ng/ml FNDC5 (Abnova), 200 ng/ml FNDC5 (Phoenix) and 60 ng/ml irisin (Phoenix). (A) Relative gene expression of *PPARγ, UCP1, TCF21* and *ZIC1* was measured by qRT-PCR after 12–14 days of differentiation. All expression data were normalized to actin; n≥4; ***p<0.001. (B) *PGC1β* and *CYCS* mRNA expression was assessed by using microfluidic card TaqMan gene expression assay, n ≥4, *p<0.05, **p<0.01. (C) Relative gene expression of *CD137* was measured by qRT-PCR on day 0 of differentiation; n = 12; ***p<0.001. (D) The increase of *UCP1* and *PPARγ* expression in six individual donors was compared after BMP7 and FNDC5 (Abnova) incubation, respectively. Preadipocytes with high CD137 expression showed a more robust activation of *UCP1* compared to *PPARγ* after BMP7 incubation. (E) Cell lysates were analysed by immunodetection using an oxidative phosphorylation antibody cocktail. A representative blot is shown. (F) Signal intensities of all complexes of the oxidative phosphorylation were quantified, summed up and normalized to ß-actin, n = 3–5, *p<0.05. (A-F) White bars, control; black bars, BMP7; horizontally hatched bar, FNDC5 (Abnova), diagonally hatched bar, FNDC5 (Phoenix); crossed bar, irisin. Data are presented as mean values ± SEM.

BMP7 potently induced a brite gene program in cultured adipocytes. Incubation with BMP7 during differentiation induced an increased expression of the general differentiation marker for adipogenesis *PPARγ* (3.6fold) (Figure 4A). Notably, *UCP1*, known as a brite marker, was even stronger enhanced (6.4 fold, Figure 4A). Additionally, the mRNA expression of *TCF21* [*20*] *,* a marker for white AT, was significantly reduced after BMP7 incubation (Figure 4A). *ZIC1* is a marker for classical brown AT of myogenic origin in mice [20] and its expression was unaltered after BMP7 incubation of human adipocytes (Figure 4A). Neither recombinant FNDC5 nor irisin had an effect on mRNA expression of *PPARγ, UCP1, TCF21* or *ZIC1* (Figure 4A).

In addition to *UCP1*, the transcription factor *PGC1β*, which regulates mitochondrial biogenesis, and *CYCS* (cytochrome c), an electron carrier protein of the mitochondrial electron transport chain, were both significantly enhanced by incubation with BMP7 (Figure 4B), while FNDC5 and irisin did not alter the mRNA level of these targets. Even higher concentrations of recombinant FNDC5 (1000 ng/ml) and irisin (600 ng/ml) as well as recombinant irisin obtained from a second company (Cayman Chemical, 60 and 600 ng/ml) had no effect on *UCP-1* and *PPARγ* mRNA level (Figure S8).

The most prominent effect on *UCP1* mRNA expression was observed in cells highly expressing *CD137*, a novel recently described marker of preadipocytes which are susceptible to browning [31] (Figure 4C, D). Our present study includes experiments with adipocytes of more than 10 different donors. Analyzing expression of *CD137* on day 0 revealed that the donors may be clustered in a CD137-low expressing and a *CD137*-high expressing group (Figure 4C). *CD137*-high expressing adipocytes were more sensitive to BMP7-induced brite differentiation, as indicated by a higher *UCP1* induction compared to the expression of the general differentiation marker *PPARγ* (Figure 4D). In marked contrast to the gene activation by BMP7, no effect of FNDC5 and irisin on classical brown and brite AT markers could be observed (Figure 4A, B). The *CD137* expression level had no impact on the FNDC5 response of adipocytes (Figure 4D). Moreover, we monitored the protein level of all four complexes of the mitochondrial respiratory chain and the ATP synthase to evaluate the results of the expression of mitochondrial target genes. Incubation of adipocytes with BMP7 during differentiation led to significantly enhanced mitochondrial protein level (1.6fold), while FNDC5 and irisin had no effect (Figure 4E, F).

**Figure 5:**
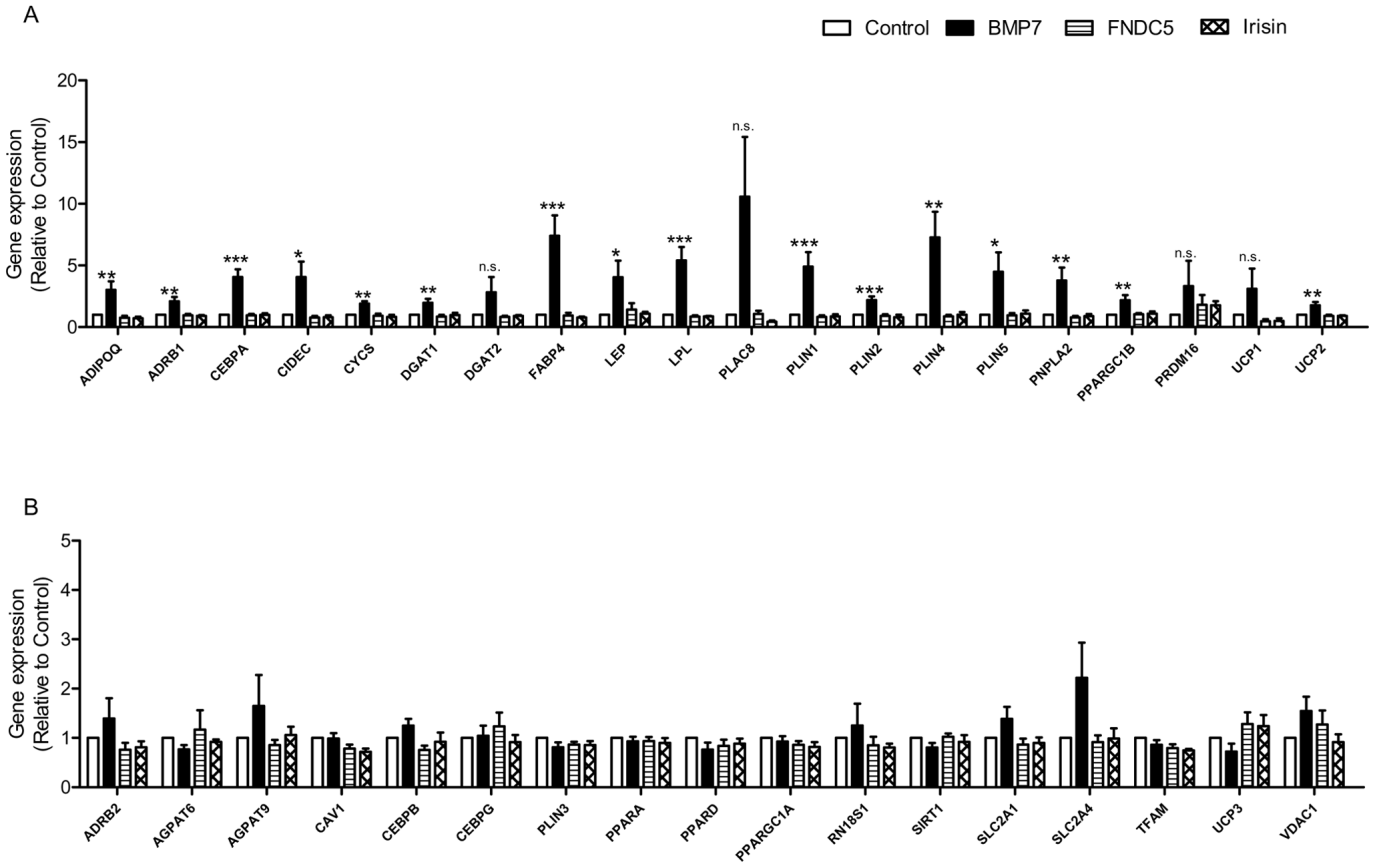
Gene expression analysis of human adipocytes after incubation with BMP7, FNDC5 and irisin. Isolated preadipocytes from human subcutaneous AT of different donors were differentiated in the presence of 50/ml BMP7, 200 ng/ml FNDC5 (Abnova), and 60 ng/ml irisin (Phoenix). Gene expression of 40 genes, related to adipocyte differentiation (A) and brite differentiation (B), was assessed by a microfluidic card TaqMan gene expression assay; n ≥4, *p<0.05, **p<0.01, ***p<0.001 vs control; n.s., not significant. White bars, control; black bars, BMP7; horizontally hatched bar, FNDC5; crossed bar, irisin. Data are presented as mean values ± SEM.

In order to assess the potential induction of genes by FNDC5 and irisin which are different from those previously measured (Figure 5), we performed a microfluidic card TaqMan gene expression assay including 37 genes associated with adipocyte differentiation or browning. Several genes were upregulated by incubation with BMP7 during differentiation including adiponectin (*ADIPOQ*), *C/EBPα, FABPB4*, leptin (*LEP*), and perilipins (*PLIN1, 2, 4* and *5*) (Figure 5A). None of these genes were differentially regulated by FNDC5 or irisin. Genes that were not regulated by BMP7, FNDC5 and irisin are presented in figure 5B.

## Discussion

Targeting irisin and its downstream signaling pathways might represent an interesting strategy to increase energy expenditure in humans and to combat obesity by inducing browning of white AT. Due to a high homology between the murine and human DNA sequence, it has been speculated that translation from the mouse model to a human therapeutic approach is possible. Boström *et al.* stated that the cleaved and released part of FNDC5, the hormone irisin, is highly conserved and identical in all mammalian species sequenced [21]. Indeed, the FNDC5 gene is well conserved between organisms with one exception reported here, namely a mutation in the start codon of the human gene.

Examining the human genomic sequence revealed that the start from UniProt entry FNDC5/Q8NAU1 (full-length protein as described by Boström *et al.* [21]) is not matched by an ATG codon and that the upstream conserved ATG of other species is mutated to an ATA codon in humans. Ivanov *et al.* performed an algorithm based analysis of the 5′-UTRs of human GenBank RefSeq mRNAs to find non-ATG start codons in humans [25]. They used sequences 5′ of the annotated start-codon and compared these to other vertebrate sequences. In this bioinformatic analysis *FNDC5* ranks high in their list, as the 5′ human amino acid sequence is almost identical to that in mouse.

Nevertheless, Kozak *et al.* have shown the presence of ATA causes low translation efficiency [26]. ATG hairpin program predicted no eligible stem-loop structure or hairpin for human *FNDC5.* These hairpin structures could increase the translation efficiency by slowing down the scanning process as helicases need time to resolve these structures and give the ribosome more time to misread the codon as an ATG start codon [27]. In higher eukaryotes non-canonical start sites are rare. A search using Uniprot and a specialized website that is based on NCBI data for annotated non-canonical ATA start sites (http://bioinfo.iitk.ac.in), resulted in only three human genes, which are translated to the protein level (Q02447, Q15561, Q99594).

Nevertheless, to challenge this bioinformatic analysis, we monitored the ability of the human transcript to be translated into protein. Overexpression of human FNDC5 in HEK293 cells with ATA as start codon provided the conclusive proof on the protein level. HEK293 cells transfected with the human expression vector using ATG as start codon produced similar amounts of full-length protein compared to mouse *FNDC5*. In contrast, the human transcript with ATA as start codon resulted in only 1% full-length protein compared to mouse FNDC5. Instead the downstream in-frame ATG (represented by the cDNA sequence NP_715637) was translated into protein. However, this ATG was used with highly reduced efficiency. Using this downstream ATG, the protein has lost the signal peptide, which leads proteins towards the secretory pathway, and almost 50% of the irisin sequence. Using an optimized expression system with a strong promoter as described here is certainly not reflecting the natural situation in human tissue. Our data support that in humans no or only very low translation of human *FNDC5* mRNA into protein is occurring and primarily a truncated version without signal peptide is produced. Consequently, irisin should not be detectable or at rather low concentrations.

Thus, we suggest that the human *FNDC5* gene might be a transcribed pseudogene that has substantially lost the ability to be translated into the full-length FNDC5 protein and possibly is unable to be processed to irisin. As a result, the mutation in the start codon of the human *FNDC5* gene may result in low translation efficiency and might explain the small release of irisin observed from primary human myotubes as compared to murine myotubes.

Physical activity promotes a more oxidative phenotype in skeletal muscle and is characterized by increased expression of *PGC1α* in skeletal muscle [29], which may enhance expression of *FNDC5* [21]. Inducing contractile activity in our *in vitro* model led to significantly enhanced secretion of the well-known myokines interleukin-6 and vascular endothelial growth factor [30]. Although using this protocol led to enhanced *PGC1α* expression in human myotubes, this did not result in a significantly enhanced *FNDC5* expression. This EPS model rather reflects a training model than acute exercise as shown by enhanced MHCI mRNA level and enhanced mitochondrial content [30]. Similar results were obtained from two different training cohorts. Neither 10 weeks of interval endurance training nor 11 weeks of strength training in healthy men resulted in increased *FNDC5* mRNA expression in skeletal muscle biopsies. However, strength training significantly upregulated the secretion of at least 11 myokines in *m. trapezius* such as plasminogen activator inhibitor 1, follistatin-like 1 and secreted protein, acidic and rich in cysteine [32]. Boström *et al.* observed enhanced *FNDC5* mRNA levels (2-fold) in a cohort of older, obese subjects after a 10-weeks protocol of endurance exercise [21]. However, using gene-chip probe sets Timmons *et al.* demonstrated that *FNDC5* induction in skeletal muscle occurred only in highly active elderly subjects compared to sedentary controls (1.3fold), which were a minority of examined subjects. Moreover, they failed to confirm increased *FNDC5* gene expression after aerobic exercise in younger subjects [33].

Another study showed that circulating irisin levels were only slightly increased (about 1.2fold) after 2 or 3 sets of double sprints after one week and not after 8 weeks of exercise [34]. However, this study measured circulating irisin levels after exercise in human plasma by using a commercially available ELISA kit. The reported irisin levels in human blood samples [34]–[37] are in conflict to our notion that human FNDC5 is not translated into full-length protein due to the non-ATG start codon. We recommend that these data has to be considered with caution and that available ELISA/EIA kits have to be reappraised by other methods e.g. mass spectrometry analysis.

Boström *et al.* [21] and Sharma *et al.* [38] used Western blot analyses to detect irisin in human and murine serum. The antibody used by the authors was obtained by Abcam and specifically detects the C-terminal region of the FNDC5 protein (the peptide used for immunization/antibody synthesis was sequenced and is highlighted in Figure 1C). FNDC5 is described as a transmembrane protein with the C-terminal tail located in the cytoplasm, whereas the extracellular N-terminal part is supposed to be cleaved and released as irisin. Thus, an antibody binding to the C-terminal region of the FNDC5 protein is unlikely to detect irisin in plasma samples.

A study with heart failure patients determined higher expression of both *PGC1α* and *FNDC5* in subjects with high aerobic performance, whereas no correlation was found in patients with low aerobic performance [39]. Nevertheless, muscle-specific overexpression of *PGC1α* in transgenic mice showed a significant increase in *FNDC5* mRNA level [21] which might suggest that a profound induction of *PGC1α* is necessary to activate the downstream target *FNDC5*. Until now, only Boström *et al*. have reported a robust activation of *FNDC5* after exercise in humans as measured by quantitative real-time PCR in skeletal muscle biopsies [21].

Exercise enhanced the appearance of putative brown adipocyte progenitor cells in brown AT [40] and was described as novel physiological stimulus for browning of visceral fat in mice after controlled treadmill running [41] and free wheel running [21]. Several lines of evidence have suggested that bone morphogenetic proteins (BMP) induce adipose cell fate determination in mammalian cells (reviewed in [42]). BMP7 specifically triggers commitment of the multipotent mesenchymal cells into the brown adipocytes lineage, inducing the expression of brown fat-specific markers such as *PRDM16* and *UCP1* [7]. Embryos of BMP7 knockout mice exhibit a marked deficiency of brown AT and nearly complete absence of UCP1 expression while adenoviral-mediated expression of BMP7 in mice results in significant increase in brown, but not in white AT and leads to an increase in energy expenditure [7]. Primary human adipocytes differentiated *in vitro* have a low basal level of *UCP1* gene expression, as described for white AT [43]. However, incubation of primary human preadipocytes with BMP7 during differentiation leads to an increase in *PPARγ* expression and an even more pronounced increase in *UCP1* and *CYCS* expression as well as enhanced mitochondrial content resulting in a brite phenotype of the adipocytes. Since *ZIC1*, a marker for classical brown adipocytes [20], was not altered by incubation with BMP7 and *PRDM16* was barely detectable, the differentiated adipocytes subjected to BMP7 incubation display no classical brown phenotype. In addition, BMP7 incubation decreased *TCF21* mRNA level, a marker for white adipocytes [20].

Wu *et al.* isolated adipose progenitor cells from murine subcutaneous white AT, immortalized the cells, generated clonal cell lines derived from single cells and analyzed the gene expression pattern of multiple cell lines after induction of differentiation and treatment with forskolin [31]. They identified a distinct pool of progenitors within white AT that can give rise to cells expressing *UCP1* upon an adequate stimulus. These brown-like or “brite” cells are similar, but not identical, to classical brown fat cells and express brite-selective genes, including a developmental *transcription* factor (*Tbx1*), a component of lipid metabolism pathways (*Slc27a1*), as well as molecules known to be important in immune und inflammatory pathways (*CD40* and *CD137*). Thus, murine brite cells have a gene expression pattern distinct from either white or brown AT. *CD137* was then used to define primary brite adipocyte precursors and *CD137*-high expressing cells showed substantially elevated expression of *UCP1* after incubation with irisin-Fc and recombinant *FNDC5* compared to *CD137*-low expressing cells [31]. In our study we observed a ‘britening’ effect of human adipocytes after incubation with BMP7 with the most prominent effect in *CD137*-high expressing cells. However, neither recombinant FNDC5 nor the cleaved protein irisin triggered a brite differentiation of adipocytes in *CD137*-high- or *CD137*-low-expressing cells. Our results are supported by data recently presented at the Annual Meeting of the American Diabetes Association by Lee et al. showing that neither FNDC5 nor irisin induces browning of human and mouse adipocytes [44].

Wu *et al.* examined the gene expression profile of brown fat from 11 adult humans and unexpectedly found that the profile was closer to that of mouse brite cells than to that of mouse classical brown cells [31]. However, the presence of classical brown AT in humans has recently been shown by three independent groups [43], [45], [46]. The gene expression of classical markers of mouse brown, brite, and white adipocytes in adult human brown AT isolated from the supraclavicular region [43] or anatomically defined neck fat [45] suggests that human brown AT might consist of both classical brown and recruitable brite adipocytes. In addition, Lidell et al. provide evidence for an anatomically distinguishable interscapular brown AT depot in human infants that consists of classical brown adipocytes [46]. When thinking about pharmaceutically targeting brown and brite AT as a therapeutic approach to counteract human obesity it is of importance to clearly identify the developmental origin of these tissues in humans. Moreover, Cannon and Nedergaard raised the question how certain white-like adipocytes, which in general possess very few mitochondria, suddenly enhance their mitochondrial complement during the britening process and from where these adipocytes originate [47]. These crucial key questions should be addressed in future studies.

In conclusion, human *FNDC5* should be annotated as a transcribed pseudo-gene that has lost the ability to be effectively translated into full-length FNDC5 protein. A shorter protein version is translated only with low efficiency, but this protein has lost the signal peptide and almost 50% of the irisin sequence. Could irisin nevertheless be a potential drug in humans if downstream regulatory pathways might still exist? We observed no effect of recombinant *FNDC5* and irisin on the britening of primary human adipocytes. Thus, we conclude that the function of irisin proposed for mice is lost in humans.

## Materials and Methods

The study to obtain biopsies from m. vastus lateralis was approved by the Regional Committee for Research Ethics, Trondheim, Norway. Written informed consent was obtained from all participants.

The study to obtain biopsies from m. trapezius was approved by the Regional Committee for Research Ethics, Oslo, Norway and written informed consent was obtained from all participants.

The procedure to obtain subcutaneous adipose tissue was approved by the ethical committee of the Heinrich-Heine-University, Düsseldorf and all the donors provided written informed consent.

### Sequence Alignment

ClustalW was used for multiple alignments. Blast searches were done using NCBI-BLAST interface. FNDC5 exon 1 sequences were obtained from ENSEMBL (Exon1_human: ENSE00001862258; Exon1_gorilla: ENSGGOE00000102667; Exon1_gibbon: ENSNLEE00000033119; Exon1_rat: ENSRNOE00000220327; Exon1_mouse: ENSMUSE00000333154. The Exon1_chimp had an gap. Therefore this gap was sequenced internally using 3 independent Chimp genomic DNAs (Exon1_chimp: ENSPTRE00000406642, ENSPTRE00000351668 and sequenced by us). All other Exon1 sequences were obtained from Ensembl. The protein sequences were obtained from Uniprot in case of the human, mouse and rat sequences (FNDC5_HUMAN_o: Q8NAU1 old version until 2012_08; FNDC5_HUMAN_c: Q8NAU1 current version since 2012_08; FNDC5_mouse: Q8K4Z2; FNDC5_rat: Q8K3V5). The gorilla and gibbon sequences were obtained from ENSEMBL (FNDC5_gorilla: ENSGGOP00000009792; FNDC5_gibbon ENSNLEP00000003686), the chimp sequence was a combination of ENSEMBL and in-house sequence. Single nucleotide polymorphism data were obtained from NCBI and ENSEMBL. UNIPROT was searched for annotated non-canonical start sites in human proteins. The search for hairpin structures close to the start codon was done with the public tool AUG_hairpin (http://wwwmgs.bionet.nsc.ru/mgs/programs/aug_hairpin/).

### Overexpression of FNDC5 Constructs in HEK293 Cells

HEK293 cells were seeded in six-well plates coated with fibronectin at a density of 4×10^5^ cells/well in DMEM, high glucose containing 10% (vol./vol.) fetal calf serum and 1× Pen/Strep 24 h prior to transfection. The HEK293 cells were transfected with human or mouse FNDC5 cloned into pcDNA5-FRT-TO_cEGFP expression vector (Figure S5). An additional vector was generated by single point mutation in the naturally occurring start codon ATA of human FNDC5 to ATG. Transfection of HEK293 cells with 2 µg DNA was done as described by the manufacturer using jetPRIME reagent (Polyplus). After 24 h cells were lysed in an ice-cold lysis buffer containing 50 mmol/l Tris/HCl (pH 7.4), 1% (vol./vol.) NP-40, 0.25% (vol./vol.) sodium-deoxycholate, 150 mmol/l NaCl, 1 mmol/l EDTA, 1 mmol/l Na_3_VO_4_, and protease inhibitor cocktail (Roche). Deglycosylation of glycoproteins in the cell lysates using PNGase F was performed as specified by the manufacturer (New England Biolabs). Samples were analysed via SDS-PAGE and immunoblotting using standard methods. Antibodies against irisin/FNDC5 were from Phoenix Pharmaceuticals and against GFP from Rockland.

### Culture of Primary Human Skeletal Muscle Cells

Human skeletal muscle cells from five healthy donors (three males, 16, 21 and 47 years old; two females, 33 and 37 years old) were supplied as proliferating myoblasts (PromoCell, Lonza and Tebu). For an individual experiment, myoblasts were seeded in six-well culture dishes at a density of 1×10^5^ cells/well and cultured to near-confluence in α-modified Eagle’s medium (αMEM)/Ham’s F-12 medium containing skeletal muscle cell growth medium supplement (PromoCell). The cells were then differentiated in αMEM containing 2% (vol/vol) horse serum (Gibco) until day 5 of differentiation and followed by overnight starvation in αMEM without serum.

### Culture of Murine C2C12 Cells

C2C12 myoblasts were seeded in six-well culture dishes at a density of 1×10^5^ cells/well and cultured to near-confluence in DMEM, high glucose containing 10% (vol./vol.) fetal calf serum (FCS). The cells were then differentiated in DMEM containing 2% (vol./vol.) horse serum until day 5 of differentiation and followed by overnight starvation in DMEM without serum.

### Detection of Irisin

Supernatants of primary human and C2C12 myotubes were collected for 24 h in serum free medium. The medium was centrifuged at 1,100 rpm for 5 min and afterwards concentrated using centrifugal filter devices with a cut off of 3 kDa (Millipore). Irisin protein levels in concentrated supernatants were quantified using EIA kit from Phoenix Pharmaceuticals according to the manufactureŕs instructions.

### Electrical Pulse Stimulation

EPS was applied to fully differentiated myotubes in six-well dishes using a C-Dish combined with a pulse generator (C-Pace 100; IonOptix). The instrument emits bipolar stimuli to the carbon electrodes of the C-dish, which are placed in the cell culture medium. The human skeletal muscle cells were stimulated (1 Hz, 2 ms, 11.5 V) for 24 h after overnight starvation in serum-free αMEM [30]. The medium was changed directly before stimulation.

### Human Interval Training Study (Endurance Training)

Skeletal muscle biopsies were obtained from a subgroup of 6 untrained men (aged 40.8±2.1 years; BMI: 26.1±1.8 kg/m^2^) before and after 10 weeks of aerobic interval training (NCT00839579) [48]. Shortly, the participants performed endurance training on a treadmill 4 × 4 min intervals at ∼90% of maximum heart frequency (HRpeak) with 3 min active recovery period at ∼70% of HRpeak between each interval, 3 times weekly. Needle biopsies of *m. vastus lateralis* of fasting subjects were obtained at least 4 days after the last training session. Total RNA was extracted using Trizol and RNeasy Mini kit (Qiagen).

### Human Strength Training Study

Seven healthy, untrained men (aged 28.3±4.2 years; BMI: 23.1±2.4 kg/m^2^) participated in a strength-training program 3 times weekly for 11 weeks [32], [49]. Before study start and at least 48 h after the last training session, needle biopsies were obtained from *m. trapezius*. Total RNA was prepared based on a modified version of the method described by Chomczynski and Sacchi [50].

### Adipocyte Culture and Immunodetection

Subcutaneous AT was obtained from healthy lean or moderately overweight women (aged 40.4±4.2 years, BMI 28.0±1.1, n = 17) undergoing plastic surgery. Preadipocytes were isolated by collagenase digestion of AT as previously described by our group [51]. Isolated cell pellets were resuspended in basal medium (DMEM/F12 medium supplemented with 14 nmol/l NaHCO_3_, 33 mmol/l biotin, 17 mmol/l D-panthothenic-acid and 10% (vol./vol.) FCS, pH 7.4), seeded in six-well plates and maintained at 37°C with 5% CO_2_. After cells were grown until confluence, cultures were washed and further incubated in an adipocyte differentiation medium (basal medium supplemented with 66 nM insulin, 1 nM triiodo-L-thyronine, 100 nM cortisol, 10 mg/ml apo-transferrin, 50 mg/ml gentamycin) for 14 days. Medium was changed every 2–3 days with addition of 5 µM troglitazone for the first three days. Adipocytes were incubated with 50 ng/ml BMP7 (R&D systems), 200 and 1000 ng/ml FNDC5 (Abnova and Phoenix), 60 and 600 ng/ml irisin (Phoenix), and 60 and 600 ng/ml irisin (Cayman Chemical) during differentiation, respectively. Immunoblotting of lysates was performed as described in [30]. Oxphos antibody cocktail was provided by MitoSciences.

### RNA-isolation and Quantitative Real-time PCR

Cells were lysed by Tripure (Roche Applied Science), RNA was isolated and reverse-transcribed using kits (RNeasy Mini, Omniscript Reverse Transcription, Qiagen) according to the manufacturer’s instructions. Gene expression was determined by quantitative real-time PCR using primers as described in Table S1 and GoTaq qPCR Master Mix (Promega) with 0.016 to 20.00 ng cDNA on a cycler (Step One Plus; Applied Biosystems). Expression of the investigated genes was normalised to actin or GAPDH and analysed via the ΔΔCt method.

For human muscle biopsy samples, total RNA was reversely transcribed into cDNA on a Gene Amp PCR 9700 thermal cycler with the High Capacity cDNA reverse Transcription kit (Applied Biosystems Foster City, CA). Quantitative real-time PCR was performed with reagents and instruments from Applied Biosystems in the 96-well format using a 7900 HT Fast instrument and the SDS 2.3 software (Applied biosystems) [52]. Predeveloped primers and probe sets (TaqMan assays, Applied Biosystems) were used to analyze mRNA levels of FNDC5 (Hs00401006_m1) and large ribosomal protein P0 (RPLP0, Hs99999902_m1). Relative target mRNA expression levels were calculated as 2^−[Ct(target)-Ct(RPLP0))]^, thereby normalizing data to endogenous control RPLP0. For expression studies in human tissues the human FNDC5 probe set Hs00401006 from Life Technologies Corporation was used. Total RNA samples from different human tissues were purchased from Clontech Laboratories, Inc.

### Microfluidic Card TaqMan Gene Expression Assay

RNA integrity was tested on an Agilent 2100 Bioanalyzer using Agilent RNA Nano chips. Only RNAs with a RIN score of 7.5 or higher were used for analysis. Synthesis of cDNA was done from 0.5 µg of each total RNA preparation in a volume of 20 µl with the Quantitect Reverse Transcription Kit from Qiagen according to the manufacturer’s instructions. Thermal cycling of the PCR reactions was done in microfluidic cards on a ViiA7 Real Time PCR 384 well cycler and fluorescence plate reader from Applied Biosystems (see Table S2 for details on used gene specific TaqMan assays).

### Statistics

Data are expressed as mean ± SEM. One-way ANOVA (post-hoc test: Tukey’s multiple comparison test) and unpaired student’s t-test were used to determine statistical significance. All statistical analyses were done using GraphPad Prism 5 considering a p-value of less than 0.05 as statistically significant.

## Supporting Information

Figure S1 **Genotyping of human FNDC5 exon 1 sequence.** Source of mRNA for 5′-RACE was (A) human skeletal muscle and (B) human cerebellum. Tissue samples were obtained from Clontech. Identified sequences with bp 55–91 of human FNDC5 variant 2 (NM_153756) and variant 3 (NM_001171940).
(TIF)

Figure S2 **Alignment of two Ref_Seq cDNAs (NM 001171940.1 and NM_153756.2) and 20 expressed sequence tags sequences.** The alignment covers the mutated start ATG to ATA codon and the CTC codon in purple that should be the start ATG, if the FNDC5 protein sequence published by Böstrom et al. [21] is forced to match the exon1 sequence.
(TIF)

Figure S3 **cDNA sequence showing the non-Kozak start ATG of NP_ 715637/NM_153756.** The 3 partial Kozak ATGs and the common stop codon (in yellow) for these 3 uORFs are boxed.
(TIF)

Figure S4 **Secondary structure of FGF2 and human FNDC5 mRNA.** Using a program for prediction of a downstream hairpin which potentially increases initiation of translation at start AUG codon in a suboptimal context showed a positive result for FGF2 (A) and no result for FNDC5 (B).
(TIF)

Figure S5 **Human FNDC5 mRNA expression levels in different human tissues.** The expression was measured by qRT-PCR and expressed relative to mRNA levels of GAPDH, shown are means +/− SD from two measurements. Total RNA samples pooled from several donors were purchased from Clontech Laboratories, Inc.
(TIF)

Figure S6 **Vectors maps of ATA-hFNDC5-GFP (A) and ATG-hFNDC5-GFP (B).**
(TIF)

Figure S7 **Quantification of GFP fluorescence in HEK293 cells.** (A) In 96 well plate format HEK293 cells were seeded at a density of 2×104/well and transiently transfected with 0.05 µg of the indicated expression vector using jetPRIME reagent. 24 h later cells were visualized using 100× magnification on an inverted fluorescence microscope. (B) Quantification of GFP signal was measured with an Ultra Evolution Tecan at 485 and 520 nm. Data are presented as mean values ± SEM.
(TIF)

Figure S8 **Isolated preadipocytes from human subcutaneous preadipocytes of different donors were differentiated in the presence of 50 ng/ml BMP7, 200 and 1000 ng/ml FNDC5 (Abnova), 60 and 600 ng/ml irisin (Phoenix) and 60 and 600 ng/ml irisin (Cayman Chemical).** (A, B) Relative gene expression of *PPARγ* (A) and *UCP1* (B) was measured by qRT-PCR after 12–14 days of differentiation. All expression data were normalized to the mRNA level of actin; n = 5–6 (for treatment with irisin provided by Cayman Chemical n = 3); ***p<0.001. (C) Cell lysates were analysed by immunodetection using an oxidative phosphorylation antibody cocktail. Signal intensities of all complexes of the oxidative phosphorylation were quantified and normalized to ß-actin, n = 3–5, **p<0.01.
(TIF)

Table S1 **Overview of used primers.**
(DOCX)

Table S2 **Gene symbols and corresponding TaqMan assay IDs provided by Applied Biosystems used for microfluidic card real-time PCR analysis.**
(DOCX)
