# Supplementary figures and images for: Evidence against a Beneficial Effect of Irisin in Humans
Source: PLoS One. 2013 Sep 11;8(9):e73680. doi: 10.1371/journal.pone.0073680 (PMC3770677; doi:10.1371/journal.pone.0073680)

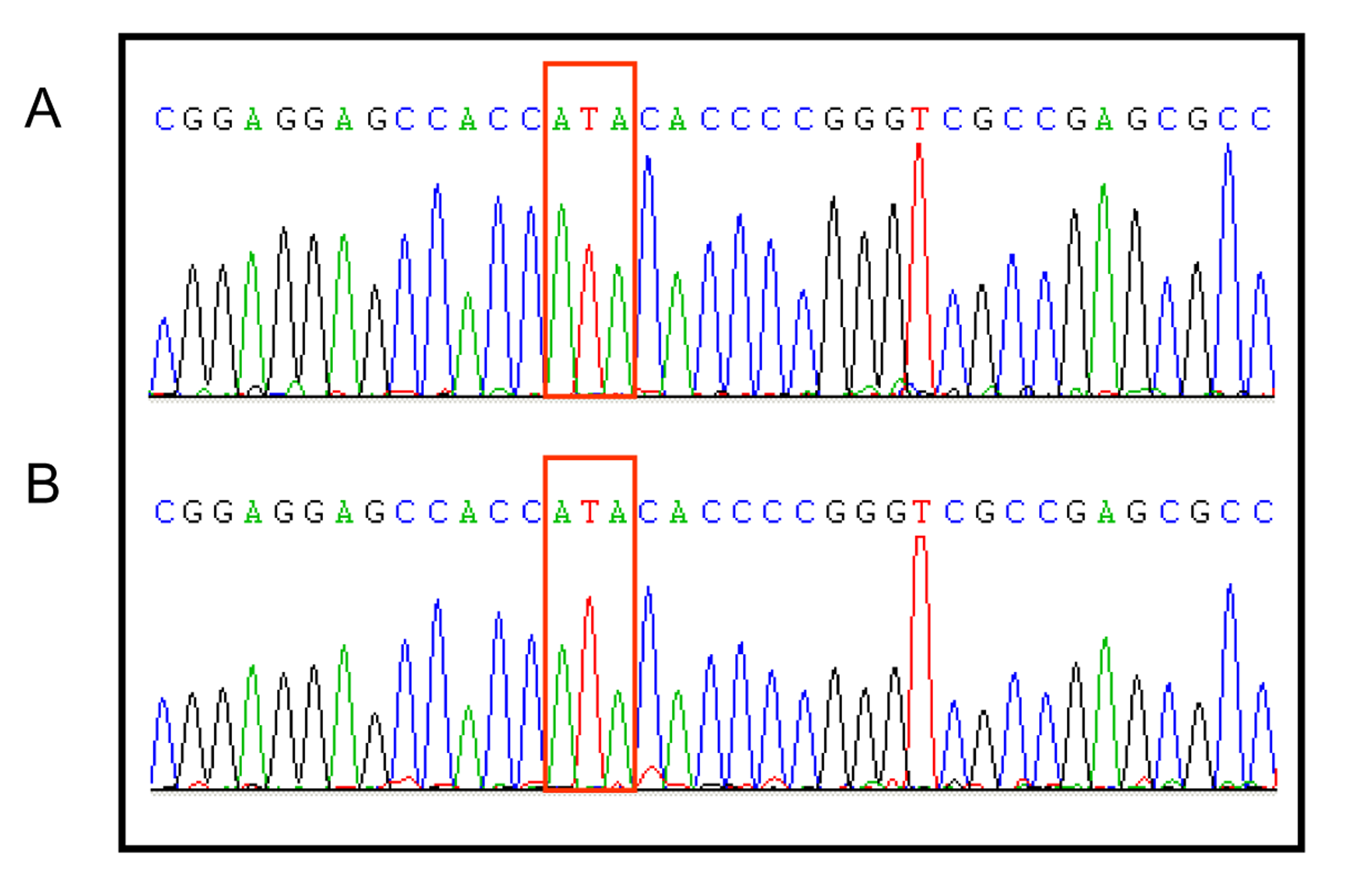

Supplement: Figure S1 — Genotyping of human FNDC5 exon 1 sequence. Source of mRNA for 5′-RACE was (A) human skeletal muscle and (B) human cerebellum. Tissue samples were obtained from Clontech. Identified sequences with bp 55–91 of human FNDC5 variant 2 (NM_153756) and variant 3 (NM_001171940). (TIF) [file pone.0073680.s001.tif]

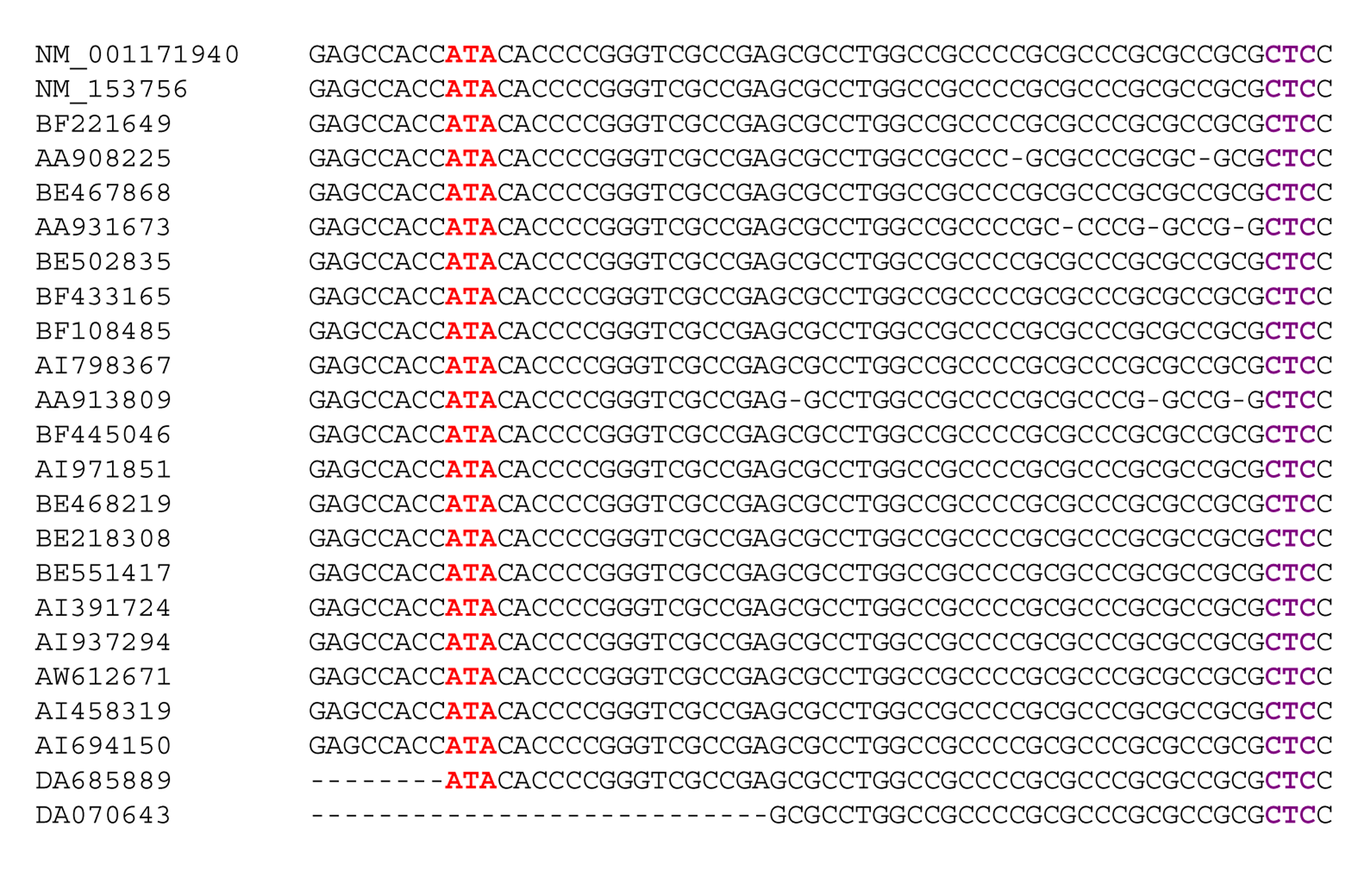

Supplement: Figure S2 — Alignment of two Ref_Seq cDNAs (NM 001171940.1 and NM_153756.2) and 20 expressed sequence tags sequences. The alignment covers the mutated start ATG to ATA codon and the CTC codon in purple that should be the start ATG, if the FNDC5 protein sequence published by Böstrom et al. [21] is forced to match the exon1 sequence. (TIF) [file pone.0073680.s002.tif]

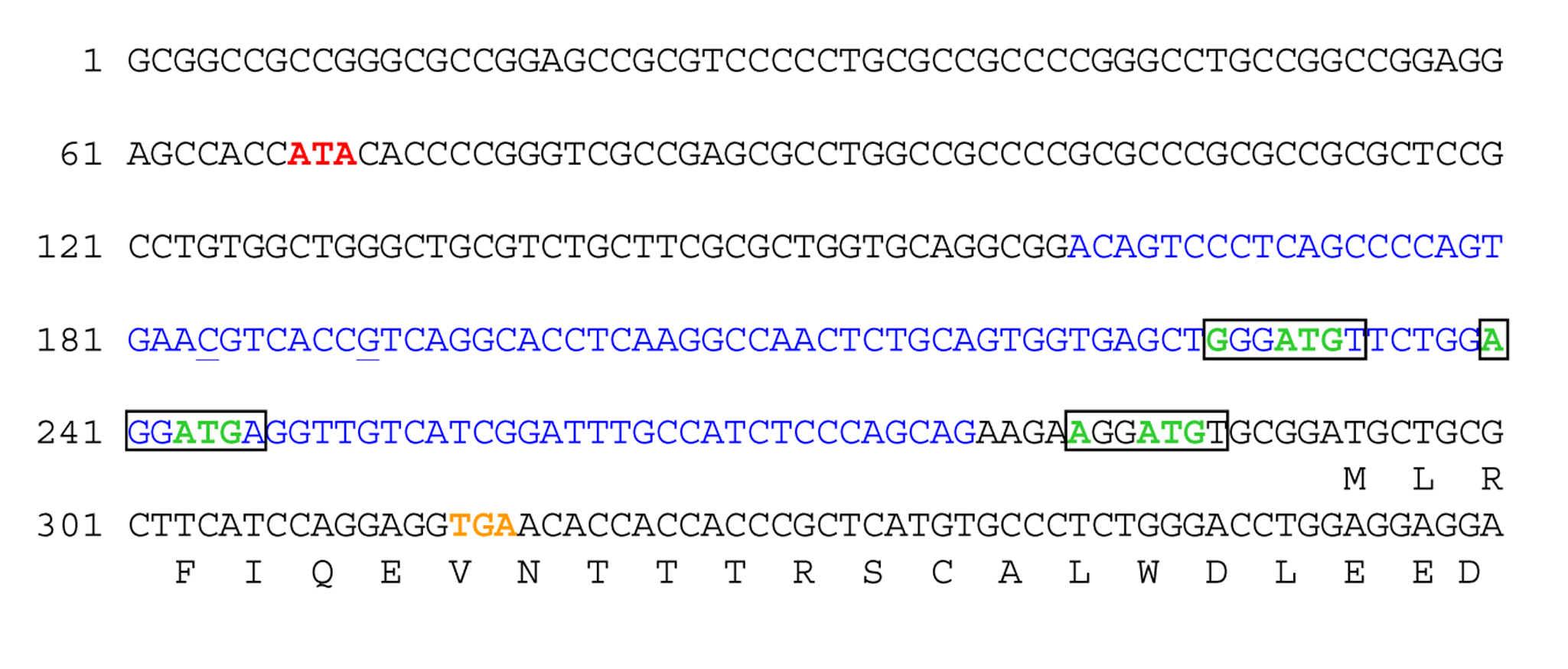

Supplement: Figure S3 — cDNA sequence showing the non-Kozak start ATG of NP_ 715637/NM_153756. The 3 partial Kozak ATGs and the common stop codon (in yellow) for these 3 uORFs are boxed. (TIF) [file pone.0073680.s003.tif]

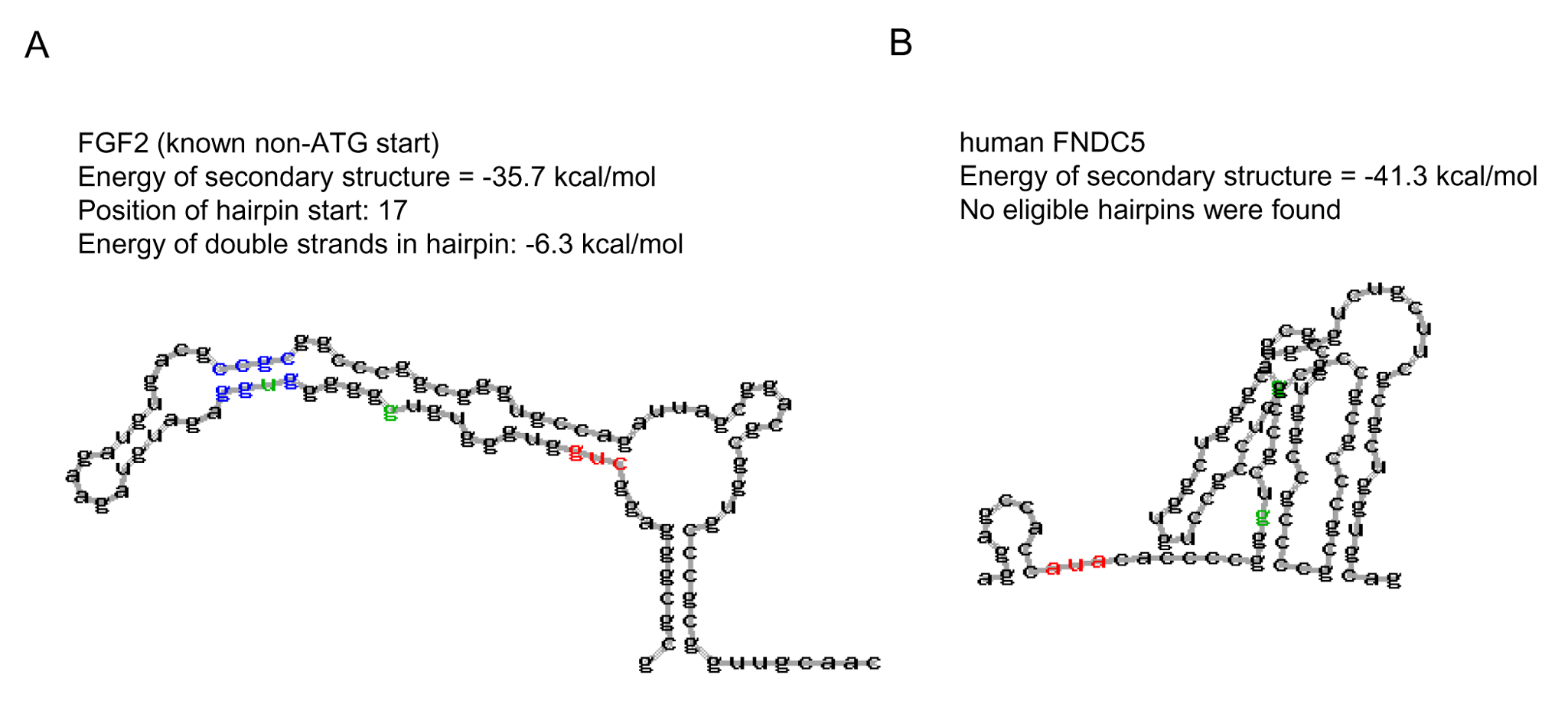

Supplement: Figure S4 — Secondary structure of FGF2 and human FNDC5 mRNA. Using a program for prediction of a downstream hairpin which potentially increases initiation of translation at start AUG codon in a suboptimal context showed a positive result for FGF2 (A) and no result for FNDC5 (B). (TIF) [file pone.0073680.s004.tif]

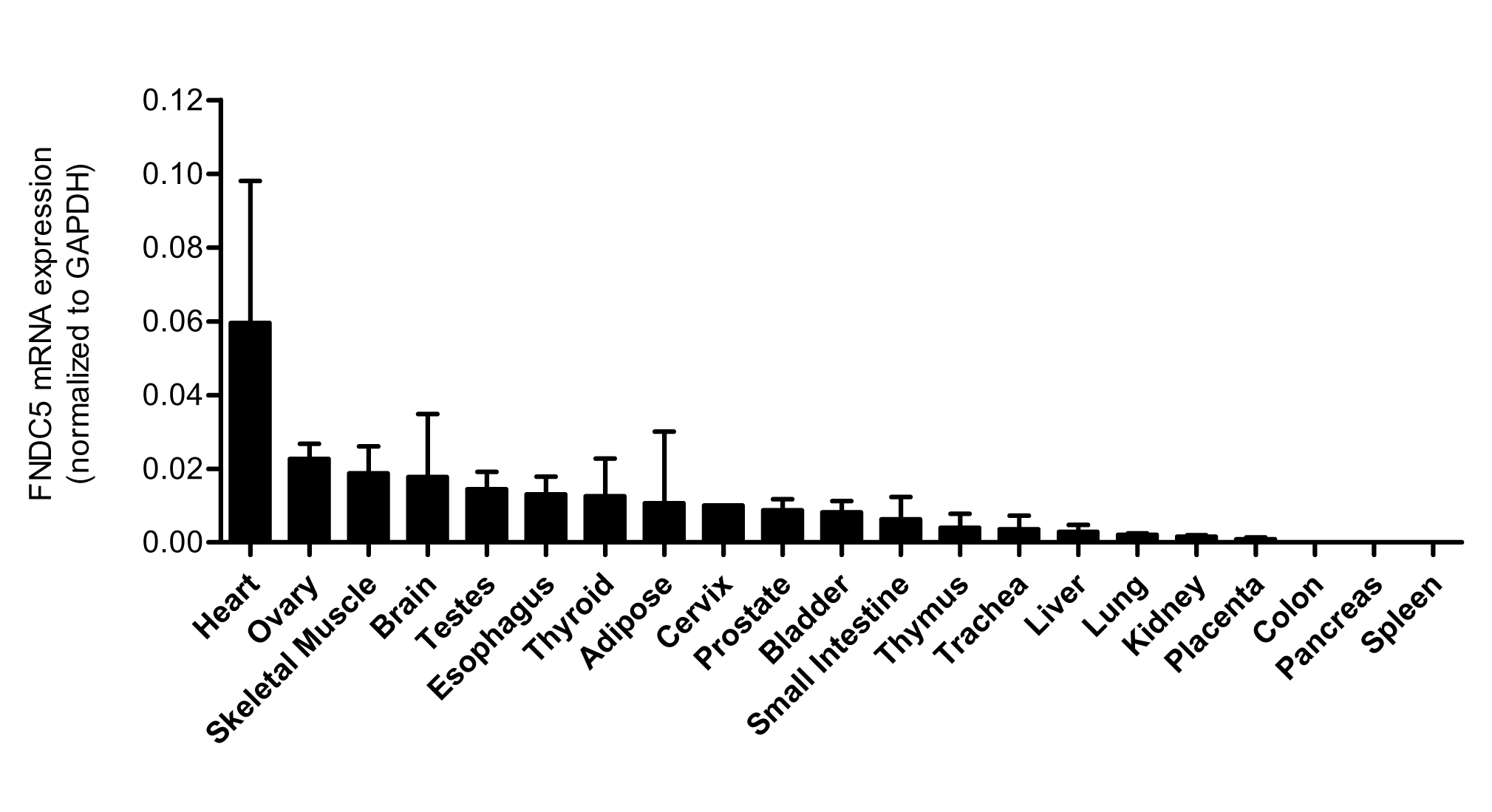

Supplement: Figure S5 — Human FNDC5 mRNA expression levels in different human tissues. The expression was measured by qRT-PCR and expressed relative to mRNA levels of GAPDH, shown are means +/− SD from two measurements. Total RNA samples pooled from several donors were purchased from Clontech Laboratories, Inc. (TIF) [file pone.0073680.s005.tif]

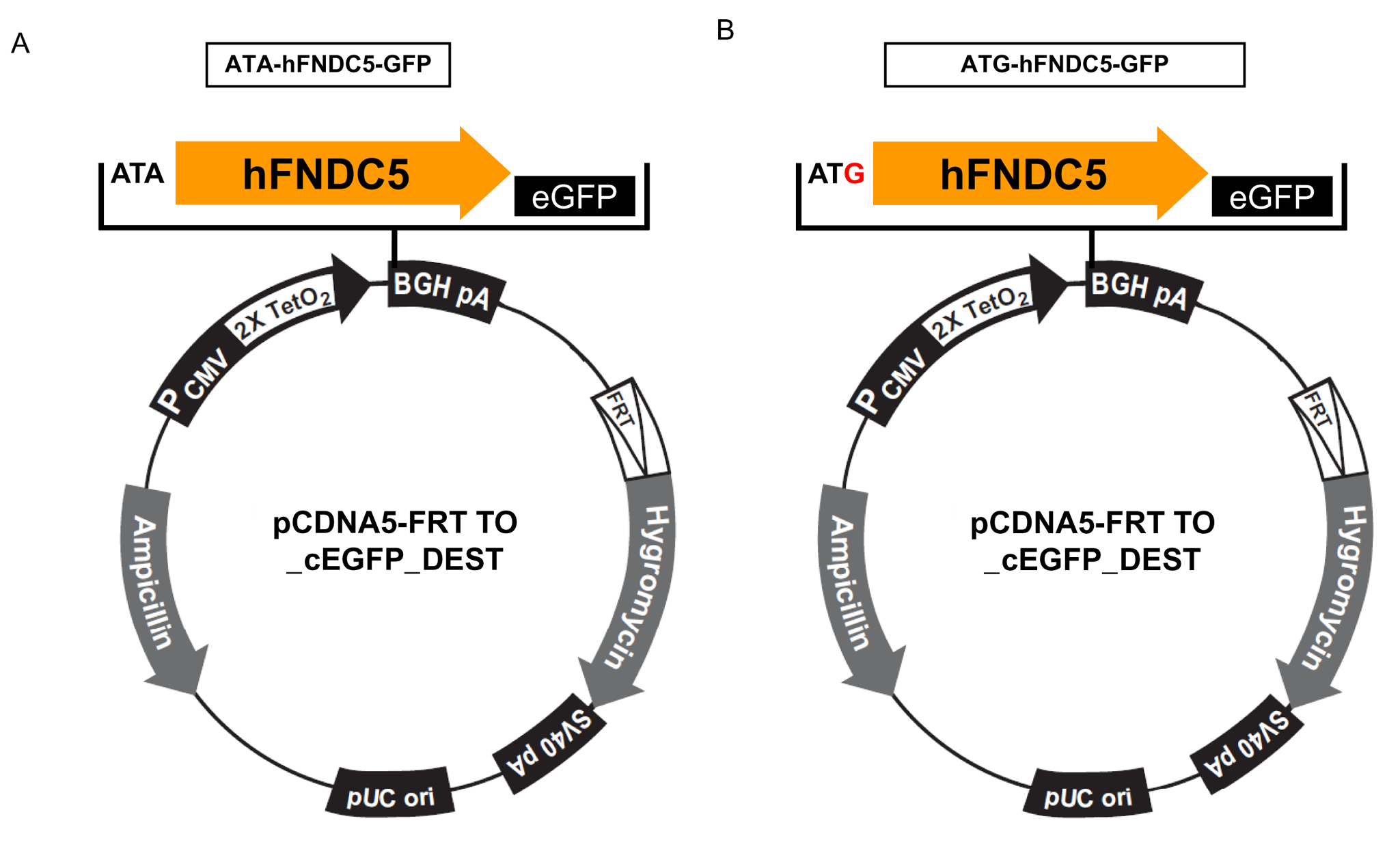

Supplement: Figure S6 — Vectors maps of ATA-hFNDC5-GFP (A) and ATG-hFNDC5-GFP (B). (TIF) [file pone.0073680.s006.tif]

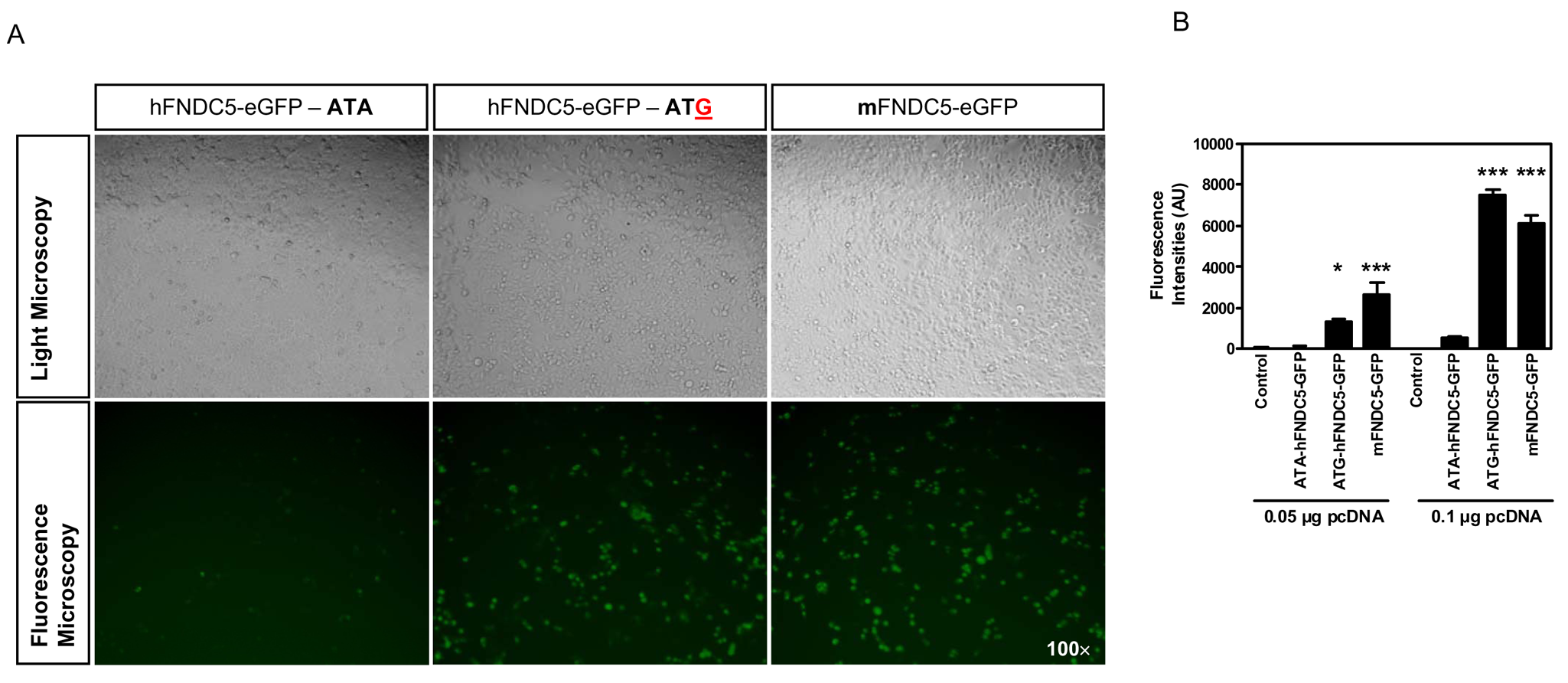

Supplement: Figure S7 — Quantification of GFP fluorescence in HEK293 cells. (A) In 96 well plate format HEK293 cells were seeded at a density of 2×10^4^/well and transiently transfected with 0.05 µg of the indicated expression vector using jetPRIME reagent. 24 h later cells were visualized using 100× magnification on an inverted fluorescence microscope. (B) Quantification of GFP signal was measured with an Ultra Evolution Tecan at 485 and 520 nm. Data are presented as mean values ± SEM. (TIF) [file pone.0073680.s007.tif]

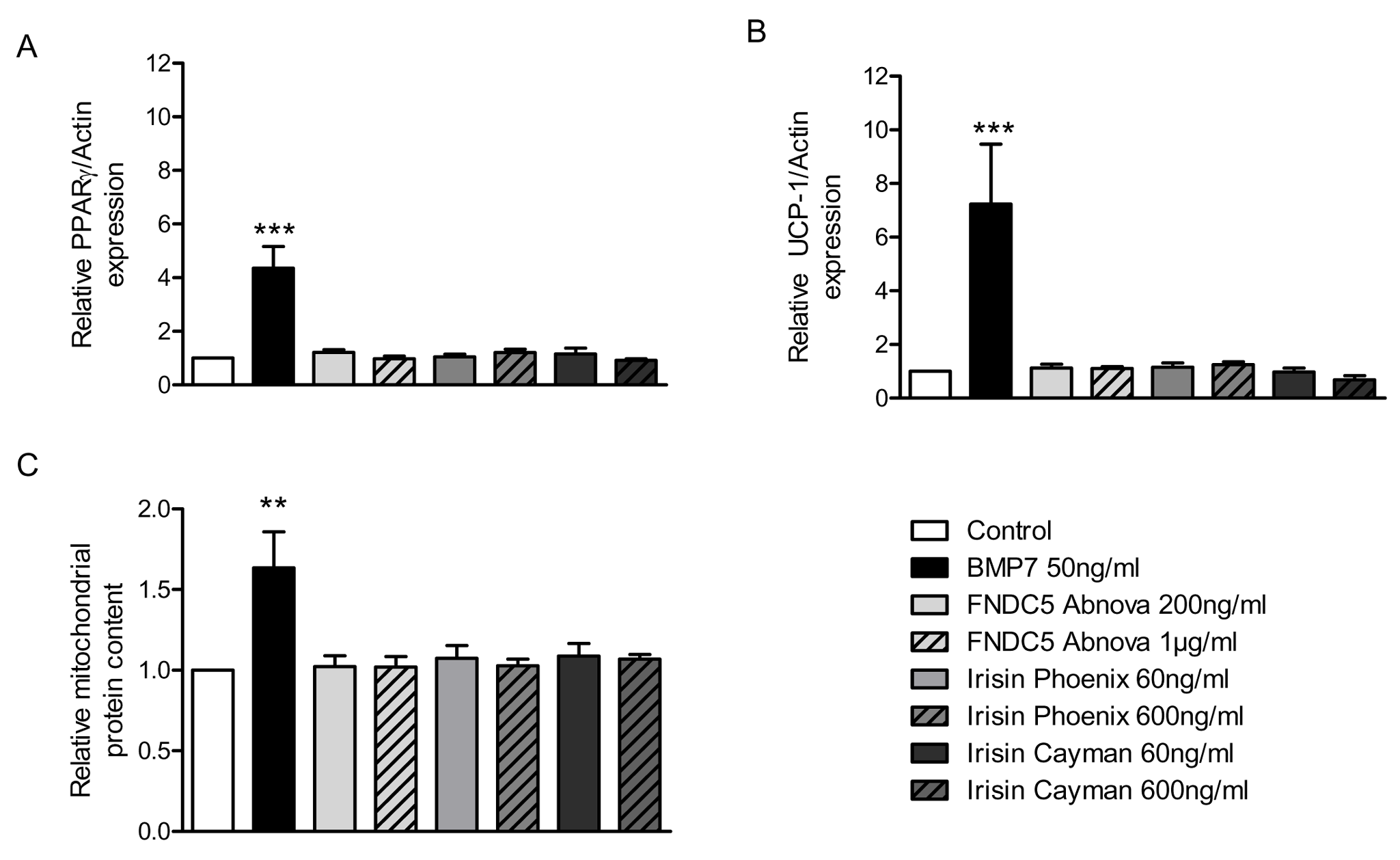

Supplement: Figure S8 — Isolated preadipocytes from human subcutaneous preadipocytes of different donors were differentiated in the presence of 50 ng/ml BMP7, 200 and 1000 ng/ml FNDC5 (Abnova), 60 and 600 ng/ml irisin (Phoenix) and 60 and 600 ng/ml irisin (Cayman Chemical). (A, B) Relative gene expression of PPARγ (A) and UCP1 (B) was measured by qRT-PCR after 12–14 days of differentiation. All expression data were normalized to the mRNA level of actin; n = 5–6 (for treatment with irisin provided by Cayman Chemical n = 3); ***p<0.001. (C) Cell lysates were analysed by immunodetection using an oxidative phosphorylation antibody cocktail. Signal intensities of all complexes of the oxidative phosphorylation were quantified and normalized to ß-actin, n = 3–5, **p<0.01. (TIF) [file pone.0073680.s008.tif]
