# Supplementary material for: Evidence against a Beneficial Effect of Irisin in Humans
Source: PLoS One. 2013 Sep 11;8(9):e73680. doi: 10.1371/journal.pone.0073680 (PMC3770677; doi:10.1371/journal.pone.0073680)
Supplement: Table S1 — Overview of used primers. (DOCX) [file pone.0073680.s009.docx]

**Table S1.** Overview of used primers.

| **Gene** | **Supplier:**  **Identifier** | **Forward Primer Sequenz (5‘→3‘)** | **Reverse Primer Sequenz (5‘→3‘)** | |
| --- | --- | --- | --- | --- |
| **Actin**  **(human)** | Qiagen:  Hs_ACTB_2_SG | Sequence not provided by supplier | | |
| **Adiponectin**  **(human)** | Qiagen: Hs_ADIPOQ_1 SG | Sequence not provided by supplier | | |
| **C/EBPα**  **(human)** | Qiagen: Hs_CEBPA_1_SG | Sequence not provided by supplier | | |
| **CD137**  **(human)** | Eurofins MWG  Operon | AGCTGTTACAACATAGTAGCCAC | TCCTGCAATGATCTTGTCCTCT | |
| **FNDC5 (human)** | Qiagen:  Hs_FNDC5_1_SG | Sequence not provided by supplier | | |
| **MYH1**  **(human)** | Eurofins MWG  Operon | CCAGACTGTGTCTGCTCTCTTCAG | | CAGGACAAGCTCATGCTCCAT |
| **MYH2**  **(human)** | Eurofins MWG  Operon | AAGGTCGGCAATGAGTATGTCA | | CAACCATCCACAGGAACATCTTC |
| **MYH7**  **(human)** | Qiagen:  Hs_MYH7_1_SG | Sequence not provided by supplier | | |
| **PGC1α (human)** | Qiagen: Hs_PPARGC1A_1 SG | Sequence not provided by supplier | | |
| **PPARγ (human)** | Qiagen:  Hs_PPARG_1 SG | Sequence not provided by supplier | | |
| **TCF21 (human)** | Qiagen: Hs_TCF21_2_SG | Sequence not provided by supplier | | |
| **UCP1 (human)** | Qiagen: Hs_UCP1_3_SG | Sequence not provided by supplier | | |
| **ZIC1 (human)** | Qiagen:  Hs_ZIC1_1_SG | Sequence not provided by supplier | | |
