# Supplementary material for: Evidence against a Beneficial Effect of Irisin in Humans
Source: PLoS One. 2013 Sep 11;8(9):e73680. doi: 10.1371/journal.pone.0073680 (PMC3770677; doi:10.1371/journal.pone.0073680)
Supplement: Table S2 — Gene symbols and corresponding TaqMan assay IDs provided by Applied Biosystems used for microfluidic card real-time PCR analysis. (DOCX) [file pone.0073680.s010.docx]

**Table S2.** Gene symbols and corresponding TaqMan assay IDs provided by Applied Biosystems used for microfluidic card real-time PCR analysis.

| **Gene Symbol** | **Assay ID** |
| --- | --- |
| ADIPOQ | Hs00605917_m1 |
| ADRB1 | Hs02330048_s1 |
| ADRB2 | Hs00240532_s1 |
| CAV1 | Hs00971716_m1 |
| CEBPA | Hs00269972_s1 |
| CEBPB | Hs00270923_s1 |
| CEBPG | Hs01922818_s1 |
| CIDEC | Hs01032998_m1 |
| CPT1A | Hs00912671_m1 |
| CYCS | Hs01588974_g1 |
| DGAT1 | Hs00201385_m1 |
| DGAT2 | Hs01045913_m1 |
| FABP4 | Hs01086177_m1 |
| LEP | Hs00174877_m1 |
| LPL | Hs00173425_m1 |
| NRF1 | Hs00192316_m1 |
| OXR1 | Hs00250562_m1 |
| PLIN1 | Hs00160173_m1 |
| PLIN2 | Hs00605340_m1 |
| PLIN3 | Hs00998421_m1 |
| PLIN4 | Hs00287411_m1 |
| PLIN5 | Hs00965990_m1 |
| PNPLA2 | Hs00386101_m1 |
| PPARA | Hs00947539_m1 |
| PPARD | Hs04187066_g1 |
| PPARGC1A | Hs01016719_m1 |
| PPARGC1B | Hs00991677_m1 |
| PRDM16 | Hs00922674_m1 |
| RN18S1 | Hs03928985_g1 |
| SIRT1 | Hs01009005_m1 |
| SLC2A1 | Hs00892681_m1 |
| SLC2A4 | Hs00168966_m1 |
| TFAM | Hs00273372_s1 |
| UCP1 | Hs00222453_m1 |
| UCP2 | Hs01075227_m1 |
| UCP3 | Hs01106052_m1 |
| VDAC1 | Hs01631624_gH |
